# Risk factors for ocular graft-versus-host disease: A systematic review and meta-analysis

**DOI:** 10.1371/journal.pone.0324703

**Published:** 2025-06-05

**Authors:** Yuanyuan Wang, Yuan Min, Yanfei Sun, Mengxin Xue, Feng Li

**Affiliations:** 1 Department of Hematology, Jinling Hospital, Affiliated Hospital of Medical School, Nanjing University, Nanjing, China; 2 The First School of Clinical Medicine, Southern Medical University, Guangzhou, China; University of Missouri, UNITED STATES OF AMERICA

## Abstract

AIM Allogeneic hematopoietic stem cell transplantation (allo-HSCT) is an important treatment for blood disease, and ocular graft-versus-host disease (oGVHD) is a common complication that significantly affects the quality of life of patients. Currently, the risk factors for oGVHD are still controversial.Methods To provide a scientific foundation for the prevention of oGVHD in patients undergoing allo-HSCT, studies on the factors influencing the development of oGVHD were searched in PubMed, Embase, Web of Science, Sino Med, the Cochrane Library, CIKI, the Wanfang Database, and the VIP Database from database construction to May 2024.Results Seventeen studies included 4,501 patients who received allo-HSCT, of which 1,526 were diagnosed with oGVHD, involving 22 factors. The overall prevalence of oGVHD was 37.8%.[95% CI (0.294, 0.463)].The prevalence of oGVHD based on the diagnostic criteria recommended by the National Institutes of Health was 46.7% [95% CI (0.390, 0.545)], that of the International Chronic oGVHD Group was 33.7% [95% CI (0.167, 0.506)], and that of self-defined diagnostic criteria was 32.0% [95% CI (0.184, 0.457)]. According to the meta-analysis, elderly patients [OR=1.10, 95% CI (1.00, 1.20)], female donors [OR=1.48, 95% CI (1.20, 1.83)], matched-relative donors (MRD) [OR=1.50, 95% CI (1.22, 1.83)], peripheral hematopoietic stem cells (PBSCs) [OR=1.81, 95% CI (1.38, 2.39)], acute graft-versus-host disease (aGVHD) [OR=1.74, 95% CI (1.29, 2.35)], chronic graft-versus-host disease (cGVHD) [OR=3.04, 95% CI (1.82, 5.08)], oral [OR=13.83, 95% CI (5.09, 37.56)] and skin graft-versus-host disease (GVHD) [OR=5.55, 95% CI (2.41,12.79)] were risk factors for the development of oGVHD (P < 0.0 5).Conclusion According to our systematic review and meta-analysis, the factors listed above are associated with oGVHD and can serve as early warning signs for clinicians in identifying high-risk populations eligible for early intervention and treatment.

## Introduction

Allogeneic hematopoietic stem cell transplantation (allo-HSCT) is an important treatment for blood system diseases. By the end of 2023, there would have been 14,551 transplant patients in China, with an 88% transplant success rate, indicating an increasing trend, according to statistics [1]. The most prevalent side effect following allo-HSCT is graft-versus-host disease (GVHD), which can be acute or chronic [2,3]. GVHD has become more common as allo-HSCT technology has been used and developed. The incidence of GVHD is estimated to be between 30% and 70%. Ocular graft-versus-host disease (oGVHD) develops in 60–90% of patients following allo-HSCT. It typically manifests as part of chronic GVHD (cGVHD) [4], occurs less frequently in acute graft-versus-host disease (aGVHD), and is associated with poor overall survival in patients after transplantation. The hallmark symptom of oGVHD is T-cell-mediated inflammatory damage, which results in dry eye (DE), cicatricial conjunctivitis, and dry keratoconjunctivitis [5]. The majority of these symptoms are accompanied by lifelong visual impairment. Patients’ everyday activities and quality of life are severely impacted by these irreversible visual impairments [2,6]. oGVHD merits greater consideration in light of the growing societal focus on transplant recipients' quality of life [7,8].

The causes of oGVHD are not widely discussed at present, and the findings of the scant published research are contradictory [9]. Meta-analysis constitutes a cornerstone methodology in evidence-based medicine, employing quantitative statistical techniques to systematically synthesize and evaluate outcomes from multiple independent studies addressing congruent research objectives. This analytical paradigm transforms fragmented empirical observations into clinically actionable high-level evidence while resolving epistemic discrepancies through quantitative synthesis [10]. Thus, the purpose of this study was to explore the risk factors for oGVHD via a systematic review and meta-analysis to identify and screen high-risk patients early and reduce the occurrence of oGVHD.

## Materials and methods

### Databases and search strategy

As required by the PRISMA declaration, our investigation was appropriately registered on the PROSPERO portal (CRD42024566501). The search strategy was developed on the basis of the following research content: (1) terms related to graft-versus-host disease or ocular graft-versus-host disease; (2) terms related to the eye; and (3) terms related to risk factors, in the form of a combination of subject terms and free words, and the finalized search terms were graft-versus-host disease/dry-eye syndrome/risk factors. Searches were performed on PubMed, Embase, Web of Science, Sino Med, the Cochrane Library, CIKI, the Wanfang Database, and the VIP Database. The initial search was conducted on May 10, 2024, and the PubMed search strategy is shown in Table 1.

**Table 1:** Strategy for searching the literature in the PubMed database.

Number	Retrieval strategy
#1	(Dry Eye Syndromes [MeSH]) OR (Ocular [All Fields]) OR (ocular surface [All Fields])
#2	(Graft vs Host Disease[MeSH]) OR (Chronic graft-versus-host disease[MeSH]) OR (Acute graft-versus-host disease[MeSH]) OR (GVHD[All Fields]) OR (cGVHD[All Fields]) OR (aGVHD[All Fields]) OR (ocular GVHD[All Fields]) OR (OGVHD[All Fields])
#3	(risk factors[MeSH]) OR (associated factors[All Fields]) OR (relative risk[All Fields]) OR (odds[All Fields]) OR (probability[All Fields]) OR (prevalence[All Fields]) OR (odds ratio[All Fields])
#4	#1 AND #2 AND #3

### Data extraction and quality assessment

Two researchers independently read all titles and abstracts and screened the literature using the inclusion/exclusion criteria. The inclusion criteria were as follows: (1) a clear definition of oGVHD; (2) oGVHD as an outcome indicator and a discussion of its influencing factors; and (3) all the original quantitative studies were considered. The exclusion criteria were as follows: (1) animal experiments; (2) conference papers or reviews; (3) non-Chinese or English literature; (4) did not extract valid data; and (5) publication time >20 years. The included literature was read in its entirety, with information retrieved, such as authors, year, region, sample size, diagnostic criteria, influencing factors, effect values, and confidence intervals. If a consensus could not be reached, it was confirmed by a third researcher.

Cohort studies were evaluated for quality via the Newcastle‒Ottawa Scale (NOS). The NOS scale is divided into 3 dimensions with 8 items. Low-risk entries are assigned 1 star up to a maximum of 9 stars, and 0--3, 4--6, and 7--9 stars are considered low-, medium- and high-quality studies, respectively [11]. Cross-sectional studies were evaluated for quality via the American Agency for Healthcare Quality and Research (AHRQ) scale, which consists of 11 items. Each entry can be answered with “yes” or “no”, with “yes” receiving one point and “no” or “unclear” receiving no points. A score of 11 was assigned, with 0--3, 4--7, and 8--11 points for low-, medium-, and high-quality studies, respectively [12].

### Statistical methods

Stata 16.0 was utilized for the statistical analysis. The effect markers for dichotomous variables were the odds ratio (OR) or hazard ratio (HR) and the 95% confidence interval (CI). Heterogeneity was determined by the χ2 test; if *P* < 0.10 and *I^2^* > 50%, the studies were considered significantly heterogeneous, and a random effects model was used; otherwise, a fixed effects model was used. Differences were considered statistically significant if *P* < 0.05. Sensitivity analyses were conducted by replacing the random and fixed effects models to compare the robustness of the combined results of the two models, and the “leave-one-out” method was used to exclude one study from each of the influencing factors of >3 included studies to reassess the change in the overall effect. According to the recommendations of the Cochrane Evidence-Based Guidelines, for fewer than 10 included studies, funnel plots were used to visually assess publication bias. For subgroups containing 10 or more studies, publication bias was statistically evaluated using Egger's linear regression test, with the significance level set at *P* < 0.05.

## Results

### Literature screening

**Fig 1:**
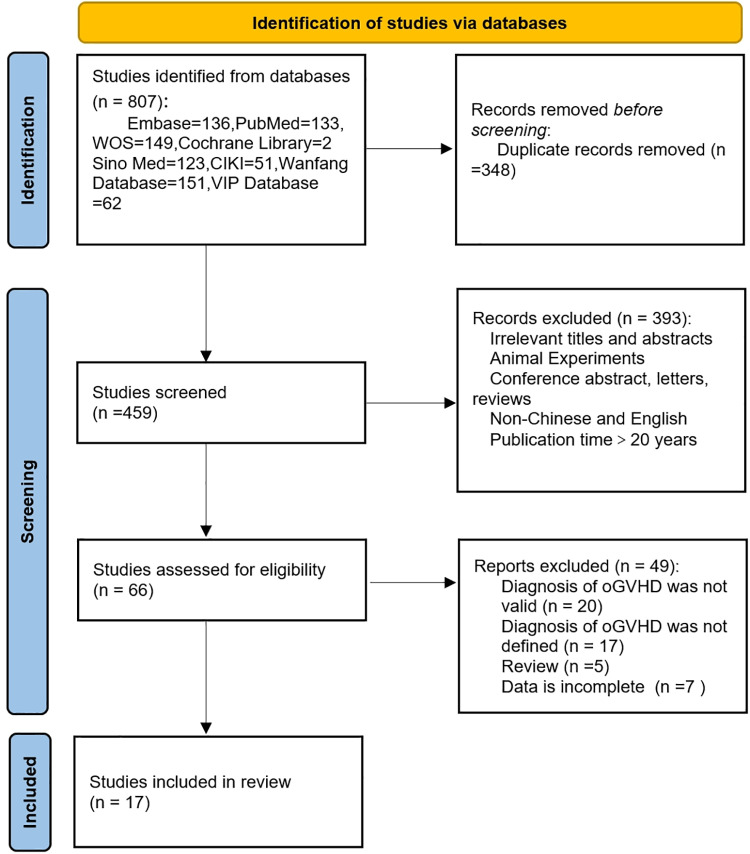
PRISMA 2020 flow diagram for systematic reviews.

While 348 duplicates were removed from the 807 articles found in the preliminary literature search, 459 articles—175 in Chinese and 284 in English—were found in the first screening. After screening the titles and abstracts of 459 papers, 66 were selected for full-text reading, and 17 documents that satisfied the requirements were ultimately included. Fig 1 illustrates the specific screening procedure.

### Basic information and quality assessment results

The seventeen studies, comprising three prospective cohort studies, nine retrospective cohort studies, and five cross-sectional studies, are listed in Table 2 along with their respective features. Of these, six studies were of moderate quality, and eleven were of high quality; the findings of the meta-analysis were largely reliable. The quality assessment results of this systematic review are presented in Tables 3 and 4. The studies included thirteen districts with a sample size of 4,480 people, and a total of twenty-two factors associated with the occurrence of oGVHD were extracted. Owing to the different results of the studies, some factors were considered relevant in some studies and irrelevant in others, which were included in the same group for analysis. The factors numbered ①--⑯ were reported in ≥2 studies, and the remaining six factors were reported in only one study; therefore, they were not included in the meta-analysis, as described in the discussion.

**Table 2:** Basic characteristics of the included studies.

First Author	Year	District	Language	Research type	Sample size (M/F)	oGVHD^criteria^	Related factors	Irrelevant factors	Quality evaluation
Fahnehjelm [13]	2008	Sweden	English	A	60(33/27)	37^a^	①⑫	/	medium
Helene [14]	2022	Denmark	English	B	418(248/170)	35^c^	①	②③⑤⑪⑫⑬⑯	high
Helene [15]	2021	Denmark	English	B	1189(724/465)	347^c^	①②⑤⑪⑫	⑨⑬	high
K-S Na [16]	2015	Korea	English	B	635	249^b^	⑭⑰⑱⑲	/	medium
Liu [17]	2016	Taiwan	English	B	139	13^a^	⑧⑨		medium
Luigi Berchicci [18]	2018	Italy	English	C	269	124^b^/154^c^	②④⑤	/	high
Marco [4]	2021	Italy	English	B	283	141^c^	①③⑨⑪㉑	㉒	high
Hoehn [19]	2020	America	English	B	162(95/67)	51^a^	⑩⑳	/	high
Meeta Pathak [20]	2018	Norse	English	A	96(44/52)	42^b^/23^c^	⑤⑩	①④⑮	high
Hébert [21]	2021	Canada	English	B	38(22/16)	15^b^	⑨	⑭	high
R Jacobs [22]	2012	America	English	A	172(89/83)	60^b^	④⑧⑨	/	high
Rehan Khan [23]	2015	India	English	B	54	17^a^	⑦⑧⑨⑩	/	high
SC Leite [24]	2006	Brazil	English	A	124(80/44)	40^a^	①⑪⑯	/	high
Sun [25]	2015	America	English	C	342	180^b^	②⑨⑮	/	medium
Tariq Aldebasi [26]	2022	Saudi Arabia	English	B	330	28^a^	①⑬	③④	high
Westeneng [27]	2010	Netherlands	English	C	101	54^a^	⑤⑦⑧⑨	/	medium
Huang [28]	2016	China	Chinese	A	89(61/28)	63^b^	⑥	/	medium

Note: A is a cross-sectional study, B is a retrospective cohort study, and C is a prospective cohort study;

^a^is self-defined,

^b^is the National Institutes of Health (NIH) criteria, and

^c^is the International Chronic Ocular GVHD Group (ICCGVHD) criteria. ① is the age of recipients, ② is the sex of the donor, ③ is the sex of the recipients, ④ is the sex match of the recipients, ⑤ is the type of donor, and ⑥ is the match of the donor's blood type. ⑦ Oral GVHD, ⑧ cutaneous GVHD, ⑨ aGVHD, ⑩ cGVHD, ⑪ stem cell source, ⑫ malignant disease, ⑬ total body radiotherapy (TBI), ⑭ diabetes mellitus, ⑮ steroid therapy, ⑯ Schirmer’s test (SIT) <10 mm/5 min, and ⑰ the number of organs with GVHD. ⑱ secondary transplantation, ⑲ ethnicity, ⑳ pretreatments with cytarabine, ㉑ pretransplant DE, ㉒ HLA matching;/indicates not mentioned.

**Table 3:** Quality assessment ratings for cross-sectional studies.

First Author	①	②	③	④	⑤	⑥	⑦	⑧	⑨	⑩	⑪	AHRQ Rating	Quality Assessment
Meeta Pathak	1	1	1	1	1	1	1	1	1	1	0	10	high
SC Leite	1	1	1	1	1	0	0	1	0	1	0	7	high
R Jacobs	1	1	1	1	1	1	1	0	0	1	0	8	high
Fahnehjelm	1	1	1	1	0	0	1	0	1	1	0	6	medium
Huang	1	1	1	1	1	0	1	0	0	1	0	7	medium

Note: ① Is the source of information (survey, literature review) clearly stated? ②Are the inclusion and exclusion criteria for exposed and non-exposed groups (cases and controls) listed or referenced to previous publications? ③ Is the time period for identifying patients given? ④ Is the study population continuous if not population-sourced? ⑤ Did the evaluators subjective factors overshadow other aspects of the study population? ⑥ Described any assessment for quality assurance (e.g., testing/retesting of subjective outcome indicators) ⑦ Explained the rationale for excluding any patients from the analysis ⑧ Described how confounding measures were evaluated and/or controlled for ⑨ Explained how missing data were handled in the analyses ⑩ Summarized the response rate of the patients and the completeness of the data collection, if there was a follow up visit, and identified the percentage of incomplete data for the expected patients or the follow up results. Percentage or follow-up results; AHRQ = Agency for Healthcare Quality and Research.

**Table 4:** Results of quality assessment of cohort studies.

First Author	Selection	Comparability	Outcome	NOS Rating	Quality Assessment
HELENE	****	**	***	9	high
HELENE	****	**	***	9	high
K-S NA	***		***	6	medium
LIU	***	*	**	6	medium
LUIGI BERCHICCI	***	*	***	7	high
MARCO	****	**	***	9	high
HOEHN	****	**	***	8	high
HÉBERT	****	*	***	7	high
REHAN KHAN	***	**	**	7	high
SUN	***		***	6	medium
TARIQ ALDEBASI	****	**	***	9	high
WESTENENG	***	*	**	6	medium

Note: NOS = Newcastle-Ottawa Scale; ① Selection: how representative was the exposed group (*), how was the non-exposed group selected (*), how were the exposure factors determined (*), identification of endpoints that were not yet available to be observed at the start of the study (*); ② Comparability: comparability of exposed and non-exposed groups was considered in the design and statistical analyses (**); ③ Outcome: adequacy of the study's evaluation of the outcome (*) whether the follow-up period after the outcome was long enough (*) and whether the follow-up was adequate in the exposed and non-exposed groups (*)

### Impact factor effect value analysis

Meta-analysis of the combined effect values of the influencing factors reported in ≥2 studies led to the conclusion that elderly age, female sex, matched-relative donor (MRD), peripheral hematopoietic stem cell (PBSC) count, and occurrence of aGVHD, cGVHD, and oral and skin GVHD were risk factors for the development of oGVHD (*P* < 0.05). The combined effect values for each factor are detailed in Table 5.

**Table 5:** Results of heterogeneity test and meta-analysis of oGVHD influencing factors.

Risk factors	Number of included studies	Heterogeneity test		Effect model	Combination OR (95%CI)		Combination Effect Quantity Test	
		*I*^*2*^ (%)	*P*		OR	95%CI	Z	*P*
**Recipient age**	8	56.80	0.013	Random	1.10	(1.00, 1.20)	2.01	**0.044**
**Donor gender**	3	19.10	0.295	Fixed	1.48	(1.20, 1.83)	3.69	**0.000**
Recipient gender	4	60.30	0.056	Random	1.55	(0.94, 2.57)	1.70	0.088
Sex matched	5	74.70	0.001	Random	0.91	(0.41, 2.01)	0.24	0.809
**Donor type**	4	0	0.581	Fixed	1.50	(1.22, 1.83)	3.89	**0.000**
Blood type matched	2	84.90	0.010	Random	0.63	(0.16, 2.50)	0.65	0.515
**aGVHD**	8	73.20	0.000	Random	1.74	(1.29, 2.35)	3.60	**0.000**
**cGVHD**	2	0	0.503	Fixed	3.04	(1.82, 5.08)	4.26	**0.000**
**Oral GVHD**	2	36.70	0.209	Fixed	13.83	(5.09, 37.56)	5.15	**0.000**
**Skin GVHD**	4	65.70	0.033	Random	5.55	(2.41, 12.79)	4.03	**0.000**
**Source of stem cells**	4	41.60	0.162	Fixed	1.81	(1.38, 2.39)	4.24	**0.000**
Malignant disease	3	73.90	0.009	Random	1.28	(0.34, 4.77)	0.37	0.711
TBI	3	0	0.521	Fixed	0.94	(0.66, 1.33)	0.37	0.712
Diabetes	2	78.30	0.032	Random	1.73	(0.25, 11.98)	0.56	0.578
Steroid therapy	2	74.30	0.048	Random	1.03	(0.21, 5.08)	0.03	0.974
SIT < 10 mm/5 min	3	83.10	0.000	Random	1.02	(0.46, 2.23)	0.04	0.968

### Prevalence of oGVHD

A total of 4,501 patients were included in this study, of whom 1,526 were diagnosed with oGVHD, and the incidence of oGVHD was 37.8% [95% CI (0.294, 0.463), *I*^*2*^ = 98.0%, *P* = 0.000], which is highly heterogeneous, so subgroup analyses were performed on the basis of diagnostic criteria. Among the seventeen studies, two provided data on both the NIH and ICCGVHD diagnostic criteria, so were included by both subgroups. Seven studies used self-defined diagnostic criteria, and the incidence of oGVHD was 32.0% [95% CI (0.184, 0.457)]. Seven studies used the NIH-recommended diagnostic criteria, and the incidence of oGVHD was 46.7% [95% CI (0.390, 0.545)]. Five studies used the diagnostic criteria recommended by the ICCGVHD, with an oGVHD incidence of 33.7% [95% CI (0.167, 0.506)]. All the subgroups presented a high degree of heterogeneity.

### Sensitivity analysis

Table 6 shows the results of using two different models for each factor. With the exception of diabetes mellitus, steroid medication, and SIT < 10 mm/5 min, all 16 factors of interest showed a high degree of concordance in the total effect sizes, suggesting that the results were typically stable. The results of the Leave-One-Out method (see S19–S30 Figs in S2 File) show that all fall within the confidence intervals.

**Table 6:** Two different models for influencing factors.

Factors	Random effects model		Fixed effects model	
	OR	95% CI	OR	95% CI
**Recipient age**	1.10	(1.00, 1.20)	1.06	(1.02, 1.10)
**Donor gender**	1.47	(1.16, 1.87)	1.48	(1.20, 1.83)
Recipient gender	1.55	(0.94, 2.57)	1.64	(1.23, 2.18)
Sex matched	0.91	(0.41, 2.01)	0.75	(0.51, 1.10)
**Donor type**	1.50	(1.22, 1.83)	1.50	(1.22, 1.83)
Blood type matched	0.63	(0.16, 2.50)	0.97	(0.66, 1.41)
**aGVHD**	1.74	(1.29, 2.35)	1.36	(1.21, 1.53)
**cGVHD**	3.04	(1.82, 5.08)	3.04	(1.82, 5.08)
**Oral GVHD**	13.09	(3.65, 46.90)	13.83	(5.09, 37.56)
**Skin GVHD**	5.55	(2.41, 12.79)	4.68	(2.92, 7.52)
**Source of stem cells**	1.95	(1.28, 2.96)	1.81	(1.38, 2.39)
Malignant disease	1.28	(0.34, 4.77)	1.12	(0.63, 2.01)
TBI	0.94	(0.66, 1.33)	0.94	(0.66, 1.33)
Diabetes	1.73	(0.25, 11.98)	2.49	(1.12, 5.55)
Steroid therapy	1.03	(0.21, 5.08)	1.54	(0.84, 2.86)
SIT < 10 mm/5 min	1.02	(0.46, 2.23)	0.58	(0.45, 0.75)

Note:/indicates that the group included ≤2 studies, not discussed.

### Bias analysis

When the incidence of oGVHD in 17 studies was examined, the results of Egger’s test revealed that there was not publication bias (t = 1.05, P = 0.308), the results are shown in S18 Fig in S2 File. And the funnel plots of factors also revealed some evidence of publication bias. Maybe attributable to notable variations across studies in temporal scope, geographical distribution, and study design, combined with insufficient sample sizes – factors likely representing the primary sources of heterogeneity.

## Discussion

The proportion of patients who survive allo-HSCT has been increasing as medicine has advanced. Even while oGVHD is not the primary cause of death for patients, it may decrease patients' quality of life and exacerbate a number of burdens from psychological and physical sources [29]. As a result, it is critical to research, assess, and mitigate the risk factors for oGVHD. The two most widely used diagnostic criteria for oGVHD are now advocated by the ICCGVHD and the NIH, as there are currently no standardized criteria for the diagnosis of this condition [30,31]. In certain preliminary investigations, researchers typically make a diagnosis. The incidence of oGVHD in this study was 46.7% according to the NIH diagnostic criteria, which is comparable to that reported in a recent cohort [18]. The current study slightly lessened the diagnostic criteria for ICCGVHD. The variability in the results may be related to the insufficient number of included studies, large differences in population characteristics, and different timings of observations.

According to a prospective study [32], age is a crucial factor to consider when transplanting. Aging increases the risk of DE and modifies the structure of the eye [33]. In this study, older patients were more likely to experience oGVHD following transplantation (*P* = 0.044). Gender also had a significant effect. These findings indicate a link between the onset of oGVHD and female donors. This may be caused by the homozygous immunological reaction that follows the identification of the Y chromosome and results in organic damage [34]. This has led some researchers to hypothesize that donor‒recipient sex matching can prevent the development of oGVHD [18]. These findings are consistent with those of earlier research on cGVHD [18,35]. However, this component was not statistically significant in this study (*P* = 0.873).

For allo-HSCT to be effective, donor T cells must undergo a graft-versus-tumor response. Since we believe that donor selection is associated with oGVHD, this study examined the donor‒recipient connection from several perspectives. The findings revealed that patients who selected MRD had a greater frequency of oGVHD than did those who selected an unrelated donor and that kinship was associated with oGVHD. This outcome can be explained by variations in the patients' pretreatment regimens and immunosuppressive medications, with patients who opt for MRD typically using milder regimens [18]. The current study, however, demonstrated that the impact of blood type incompatibility between donors and recipients progressively diminished and was not a significant predictor of oGVHD (*P* = 0.515). Bone marrow (BM), cord blood, or peripheral blood are the usual sources of donor cells. The use of PBSCs has increased dramatically as a result of their benefits over BM, which include simple collection, less cell contamination, and quicker implantation [36]. Nonetheless, the use of PBSCs increases the incidence of cGVHD [37,38]. The significantly greater concentration of T cells and CD34 + cells in the PBSC grafts than in the BM grafts may be the cause of this connection [3]. Additionally, patients who receive BM as grafts have better psychological status and prognoses, which helps them return to work [39]. The choice of PBSCs as grafts is a substantial risk factor for oGVHD, and this investigation confirms that conclusion (*P* = 0.000). Given the limited selection, the influencing factors associated with the objective conditions of the receptor supply cannot be changed or directly intervened, and the clinician can do more by paying attention to the appearance of the patient's eye symptoms and detecting oGVHD as early as possible.

Prior to receiving a donor cell transfusion, patients must be pretreated. This is a crucial tool for GVHD prophylaxis and consists of several chemotherapy regimens, the majority of which are coupled with TBI [40]. According to several theories [41], TBI damages the lacrimal gland, which can result in DE. Steroids are anti-inflammatory and pro-apoptotic, but because they can cause a variety of infectious and noninfectious problems, their prolonged use should be avoided [42]. Pretreatment regimens that minimize pretreatment-related toxicity while promoting implantation and reducing GVHD incidence have been optimized in recent years [43]. TBI and steroid therapy were not significant risk factors for oGVHD in the current investigation.

Among the studies [14,16,20,26] we analyzed, researchers discussed the impact of various conditioning regimens (e.g., immunosuppressants or chemotherapeutic agents) on oGVHD. Despite protocol variations, all studies consistently concluded that this factor showed no significant association with oGVHD.

We propose that oGVHD and systemic GVHD may share immunopathological mechanisms. Research indicates a robust correlation between the onset of oGVHD and systemic GVHD [18]. Similarly, patients with prior aGVHD (P = 0.000) and cGVHD (*P* = 0.000) had an increased risk of oGVHD in our study. When systemic GVHD occurs in patients, it indicates that the immune response may be widespread across multiple organs, including the skin, liver, gastrointestinal tract, etc. Patients with a history of oral and cutaneous GVHD are more prone to develop eyelid margin lesions [44] because the inner surface of the catheter is made of mucosa [16,45] and is frequently targeted by T cells or other inflammatory cells.

The results indicate that the likelihood of developing eyelid edge lesions is greater in both acute and chronic cases. The findings demonstrated that the development of oGVHD in both the acute and chronic phases was linked to both cutaneous (*P* = 0.000) and oral (*P* = 0.000) GVHD. Ocular surface damage is caused by gastrointestinal and hepatic GVHD, according to certain studies [45], and thus increases the likelihood of developing oGVHD. Furthermore, it has been suggested that the quantity of organs involved may play a significant role in ocular involvement [16]. This suggests that the ocular surface—a region with weaker immune privilege—is more vulnerable to attack, as systemic immune reactions can directly target this site. Ocular pathological changes represent a local manifestation of systemic immune dysregulation rather than isolated events. Therefore, controlling systemic GVHD is the central strategy for preventing and treating ocular complications.

Unexpectedly, oGVHD may manifest before other tissues and organs, and rejection responses to the eye and other organs may be mutually causal, mutually encouraging, and lead to malignant cycles. To prevent the onset of GVHD, we must thus provide patients with various systemic host-resistant illnesses with more care while providing effective therapy and symptom management. However, it is noteworthy that in the report by ZHUANG [45], approximately 6% of patients exhibited ocular symptoms without rejection reactions in other organs, suggesting that the development of oGVHD may precede pathological manifestations in other tissues and organs. The driving mechanisms linking systemic GVHD and oGVHD require further investigation.

There is ongoing debate concerning a few of the oGVHD-related variables. In contrast to the findings of HÉBERT et al. [21], Na et al. [16] reported that hyperglycemia affects the function of the ocular surface immune response and proposed that a history of diabetes is a risk factor for the development of oGVHD. A prospective investigation revealed that increasing the humidity level in the ward is a successful preventive strategy [46]. According to research by Jay et al. [9], being white may help prevent oGVHD. Nevertheless, more thorough evidence does not bolster these theories.

### Limitations

There are several limitations to this study. First, this field contains multiple knowledge gaps where research findings remain fragmented. There is not much prospective evidence, the study is mostly retrospective. Second, although our model demonstrates reasonable stability, variations in confounding factors may predispose the analysis toward individual-level effects rather than population-level considerations—an unavoidable bias inherent to small-sample investigations and the primary source of heterogeneity in this study. Consequently, caution is warranted when interpreting these findings. Third, standardized baseline ophthalmic examinations are generally lacking for hematologic patients, with current diagnostic practices predominantly relying on patient-reported symptoms or observable early clinical manifestations. Fourth, among the validated risk factors for oGVHD in this study are non-modifiable objective conditions, which inherently limit the clinical controllability and practical applicability of the conclusions. Furthermore, most existing studies have inadequately addressed potential pre-existing ocular comorbidities or systemic conditions in their study populations. Notably, none of the currently included studies have systematically examined these adjustable parameters. Future research should prioritize investigation of modifiable factors such as treatment protocols, pharmacological agents, and laboratory biomarkers in relation to oGVHD development.

## Conclusion

Research has revealed that the risk factors for oGVHD include the incidence of aGVHD, cGVHD, oral and skin GVHD, female donors, MRD, PBSC, and elderly patients. Through systematic meta-analysis, we achieved synthesis of existing evidence. Despite methodological limitations inherent to the restricted number of primary studies, this work successfully established a robust knowledge framework. Our findings not only provide researchers with a reference for evidence grading, but more critically, by identifying evidence gaps and proposing optimized research directions, lay a theoretical and hypothesis-driven foundation for future high-quality investigations involving large-scale cohorts and multi-center validation trials. To provide a more pertinent and rigorous scientific evidence-based clinical basis, hematologists and ophthalmologists must collaborate to perform high-quality, multicenter, large-sample studies in the future. Clinicians can create oGVHD prediction models for patient risk assessment on the basis of the findings of this study. Targeted baseline ophthalmologic examination, early identification of high-risk patients, health promotion, and follow-up were performed. Improving oGVHD-related visual loss or significant eye damage is essential for improving patient prognosis and posttransplant quality of life.

## Supporting information

S1 File List of raw analysis data. (XLSX)

S2 File Data analysis and results. (DOCX)
