# Supplementary material for: Risk factors for ocular graft-versus-host disease: A systematic review and meta-analysis
Source: PLoS One. 2025 Jun 5;20(6):e0324703. doi: 10.1371/journal.pone.0324703 (PMC12140268; doi:10.1371/journal.pone.0324703)
Supplement: S2 File — (DOCX) [file pone.0324703.s002.docx]

**Supporting information**

**S1Fig. Forest plot of the prevalence of oGVHD**

**
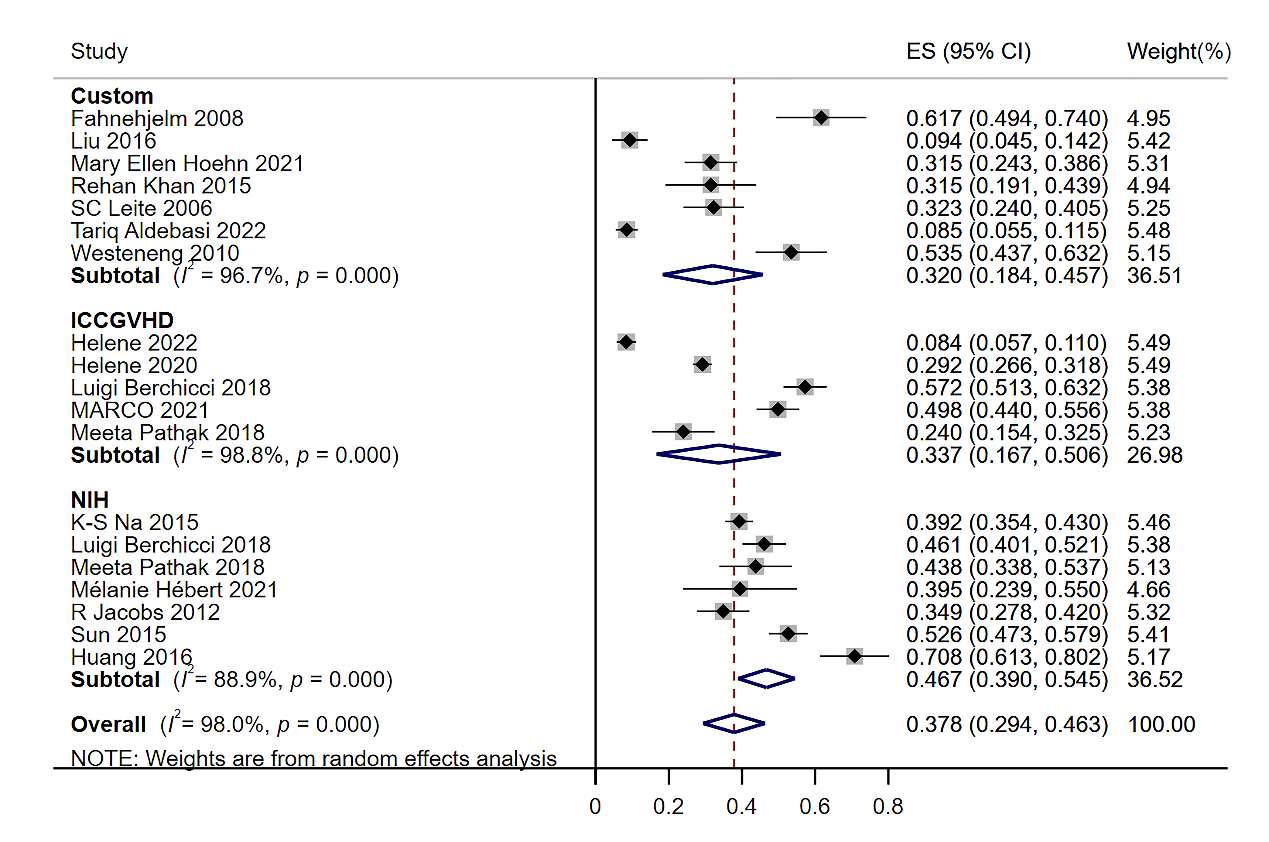
**

**
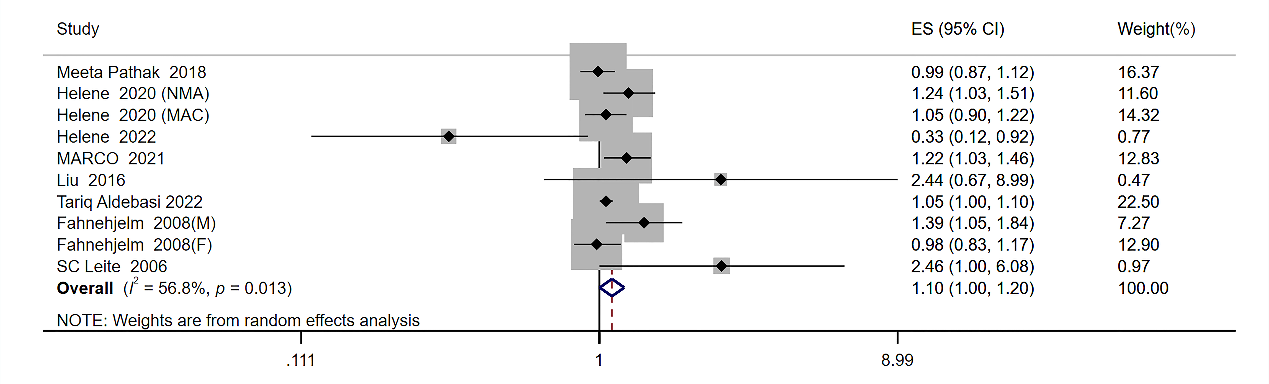
 S2Fig. Forest plot of recipient age**

**S3Fig. Forest plot of donor gender
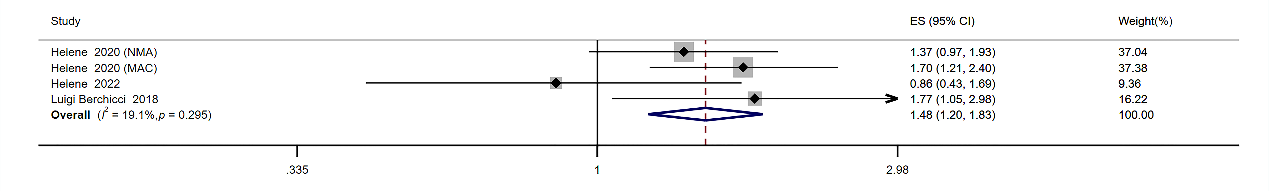
**

**S4Fig. Forest plot of
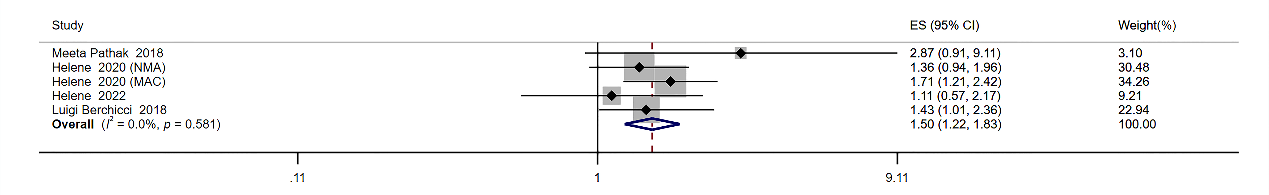
donor type**

**S5Fig. Forest plot of
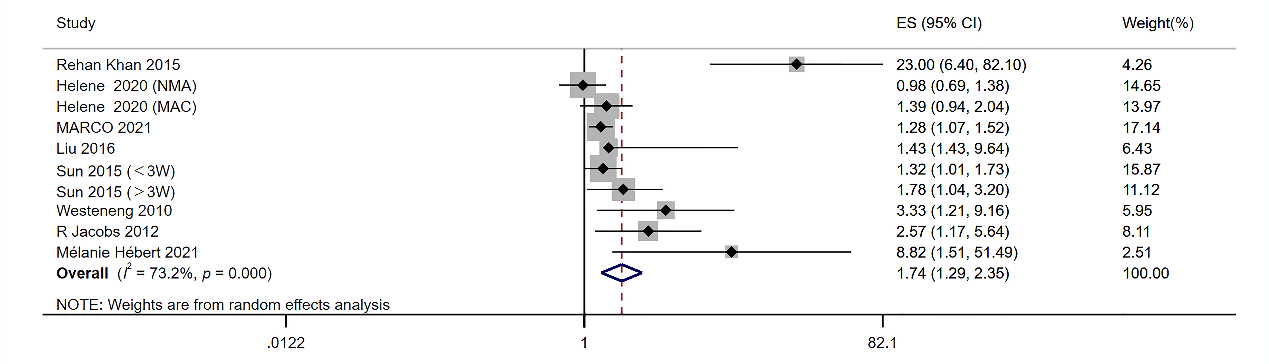
aGVHD**

**S6Fig. Forest plot
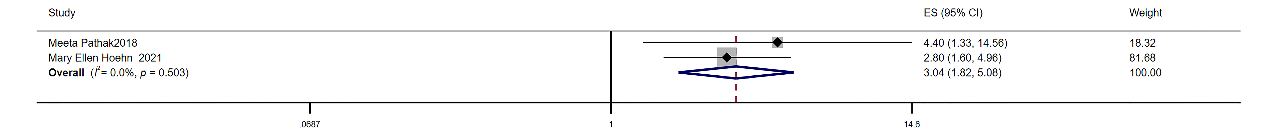
 of cGVHD**

**S7Fig. Forest plot o
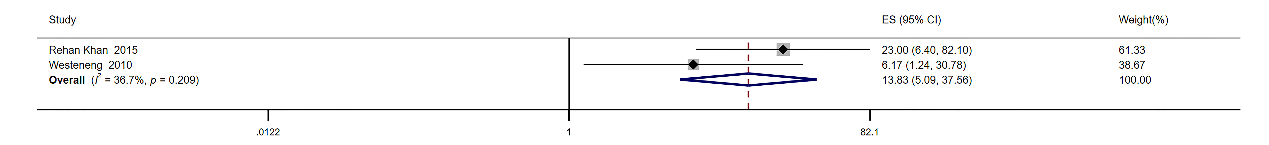
f oral GVHD**

**
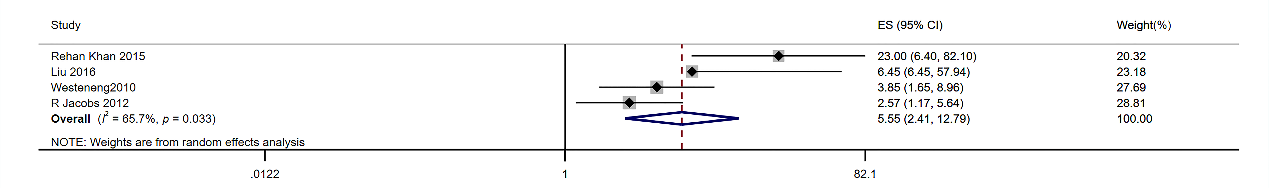
S8Fig. Forest plot of skin GVHD**

**S9Fig. Forest plot of
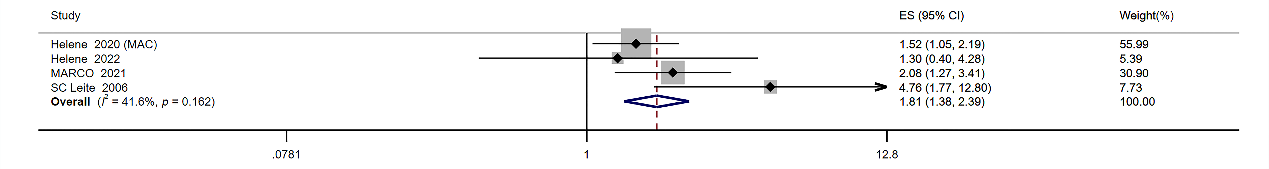
 source of stem cells**

**S10Fig. Funnel plot of recipient age**

**
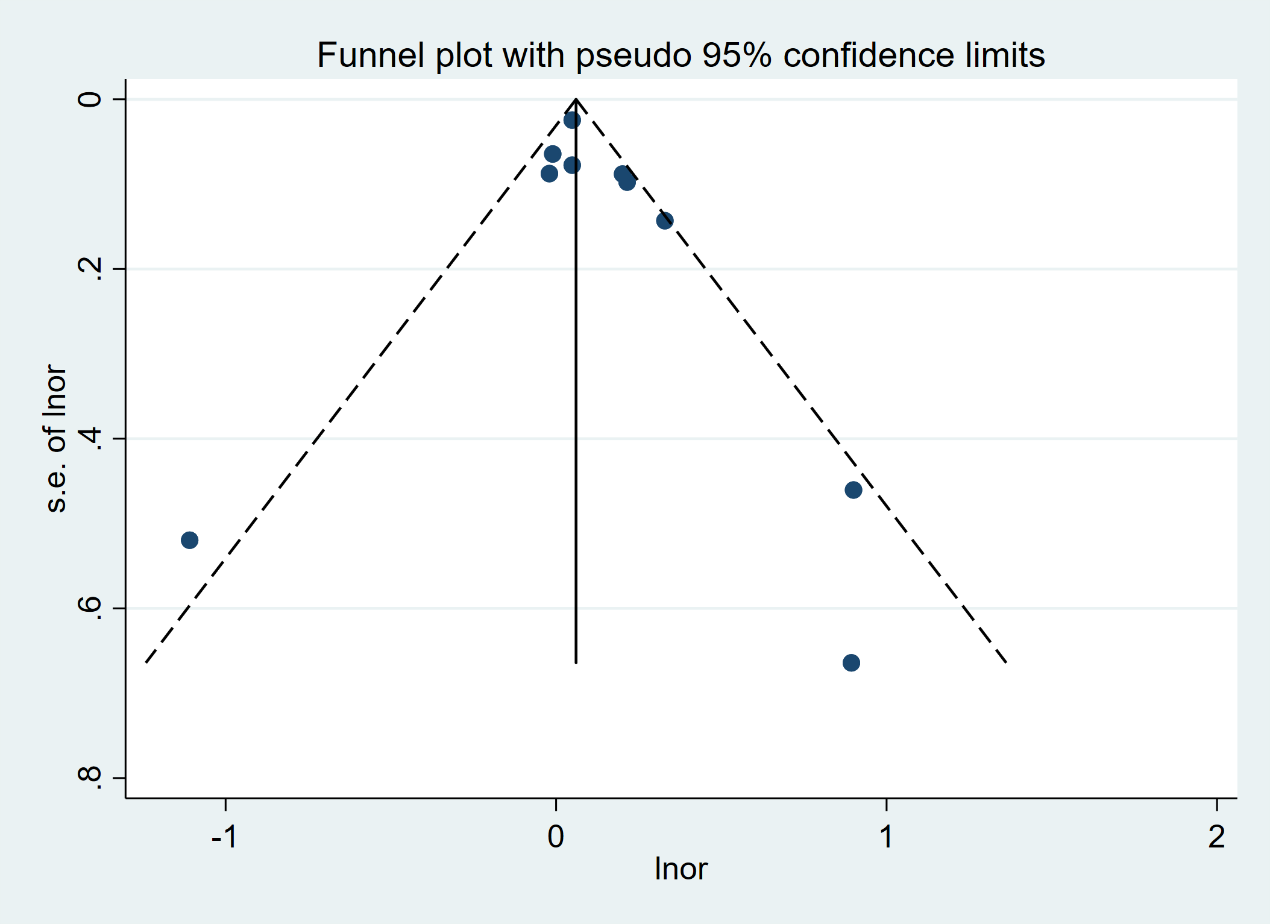
**

**S11Fig. Funnel plot of donor gender**

**
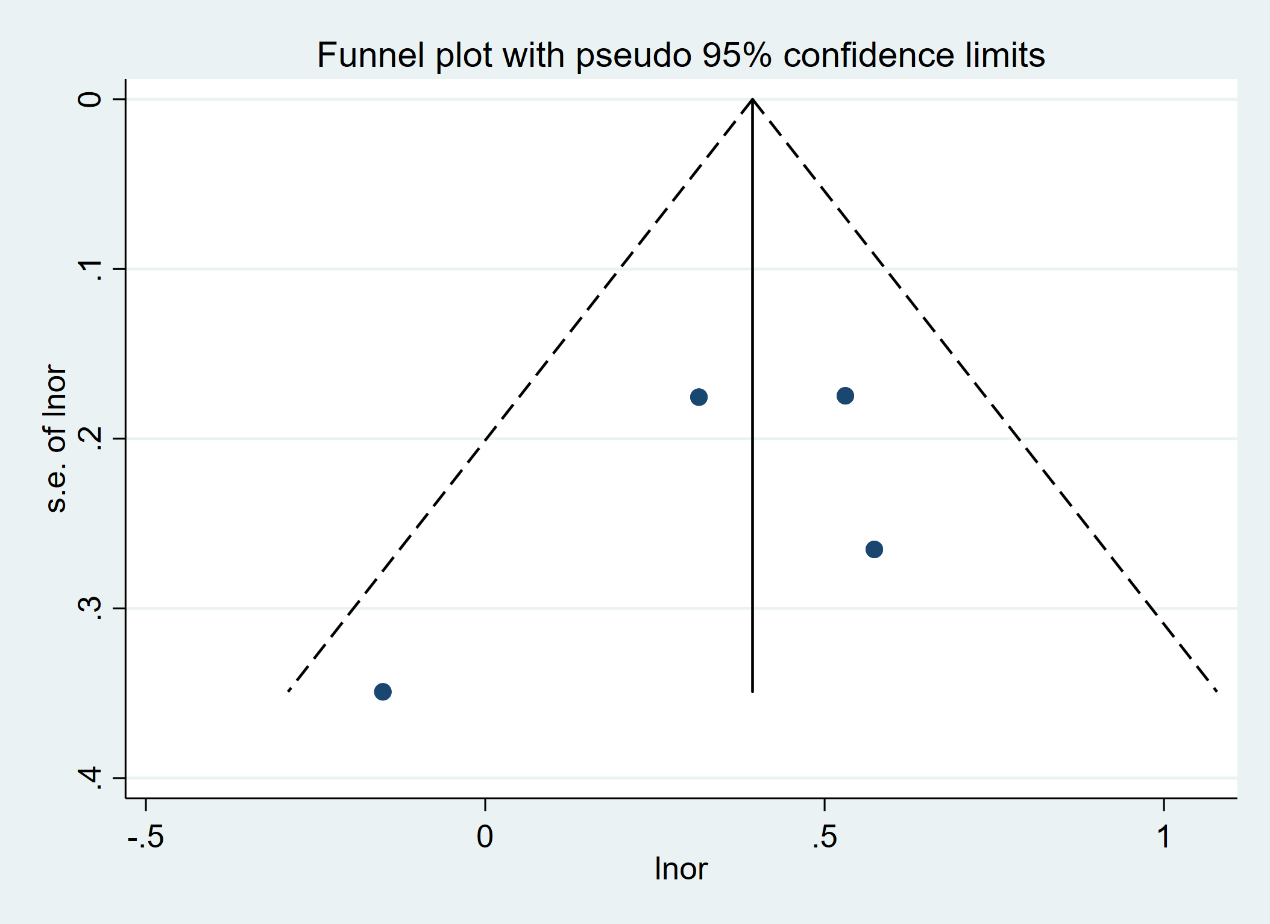
**

**S12Fig. Funnel plot of donor type**

**
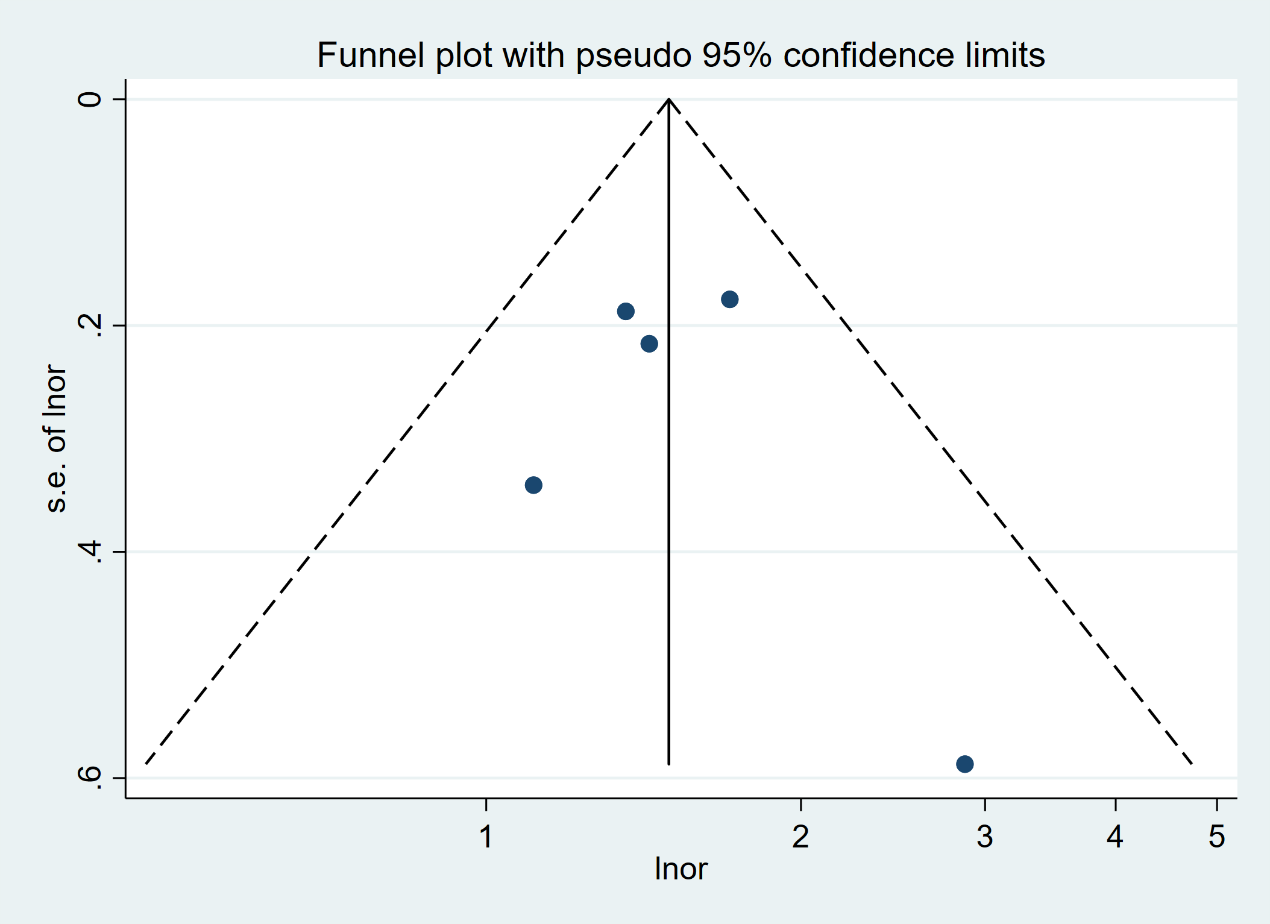
**

**S13Fig. Funnel plot of aGVHD**

**
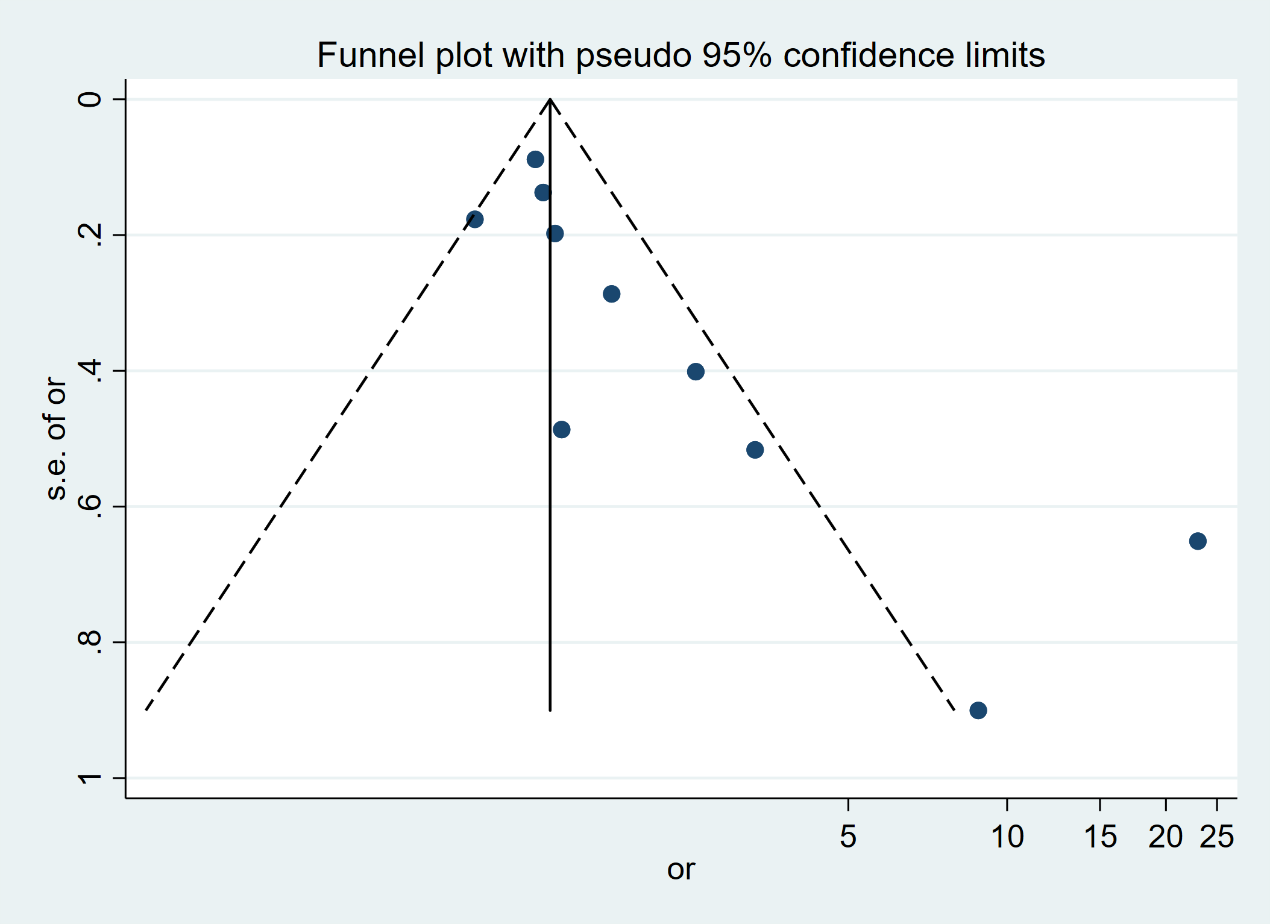
**

**S14Fig. Funnel plot of cGVHD**

**
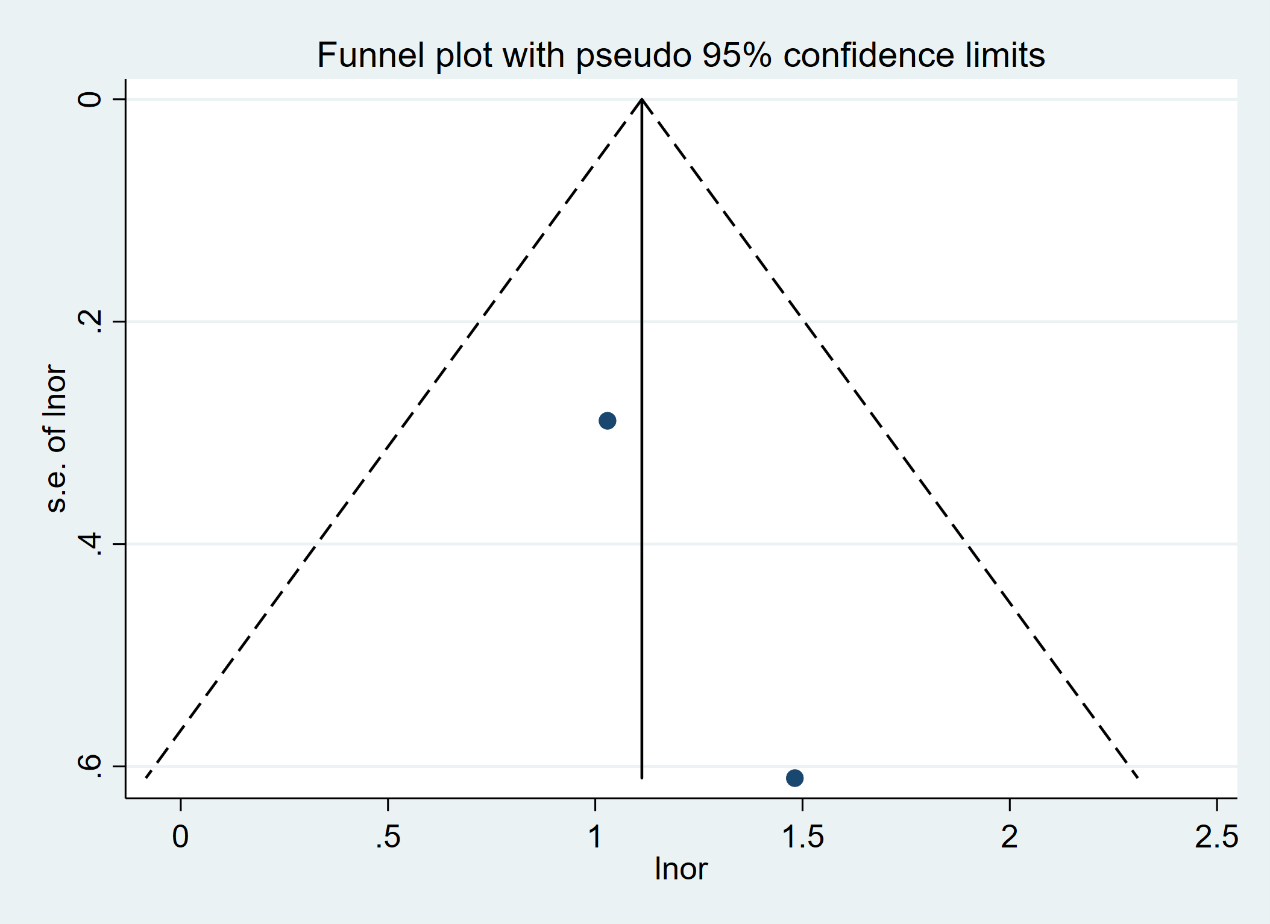
**

**S15Fig. Funnel plot of Oral GVHD**

**
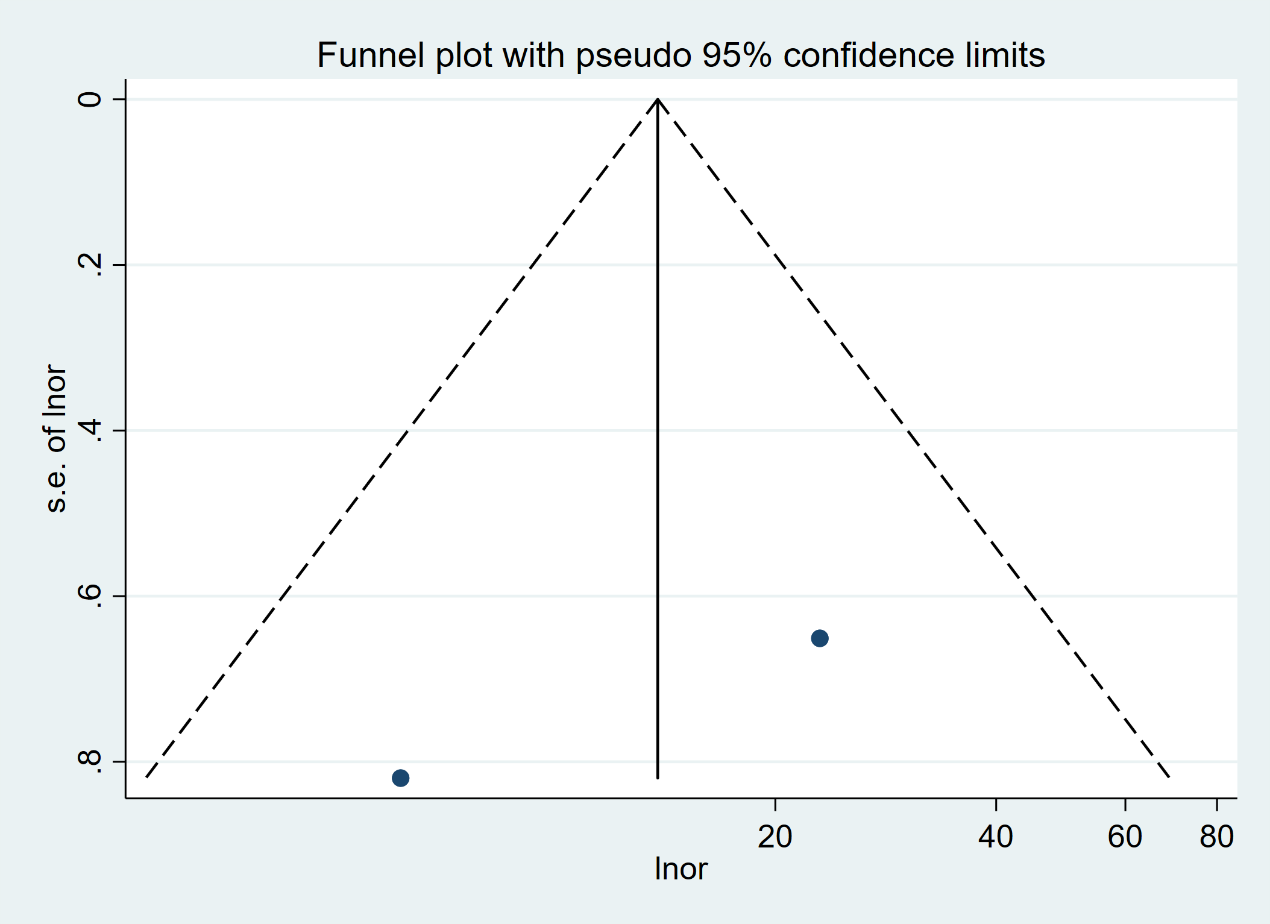
**

**S16Fig. Funnel plot of skin GVHD**

**
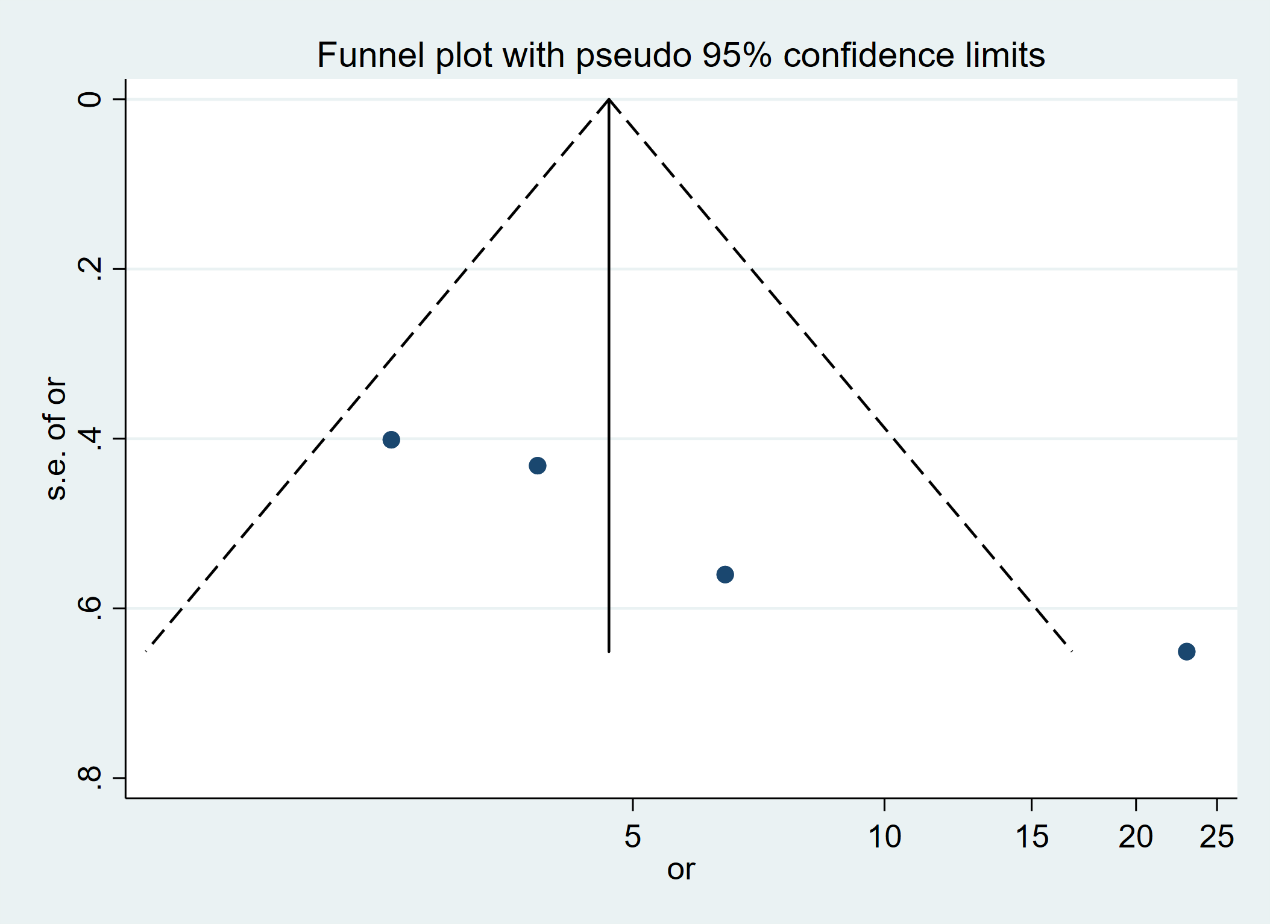
**

**S17Fig. Funnel plot of source of stem cells**

**
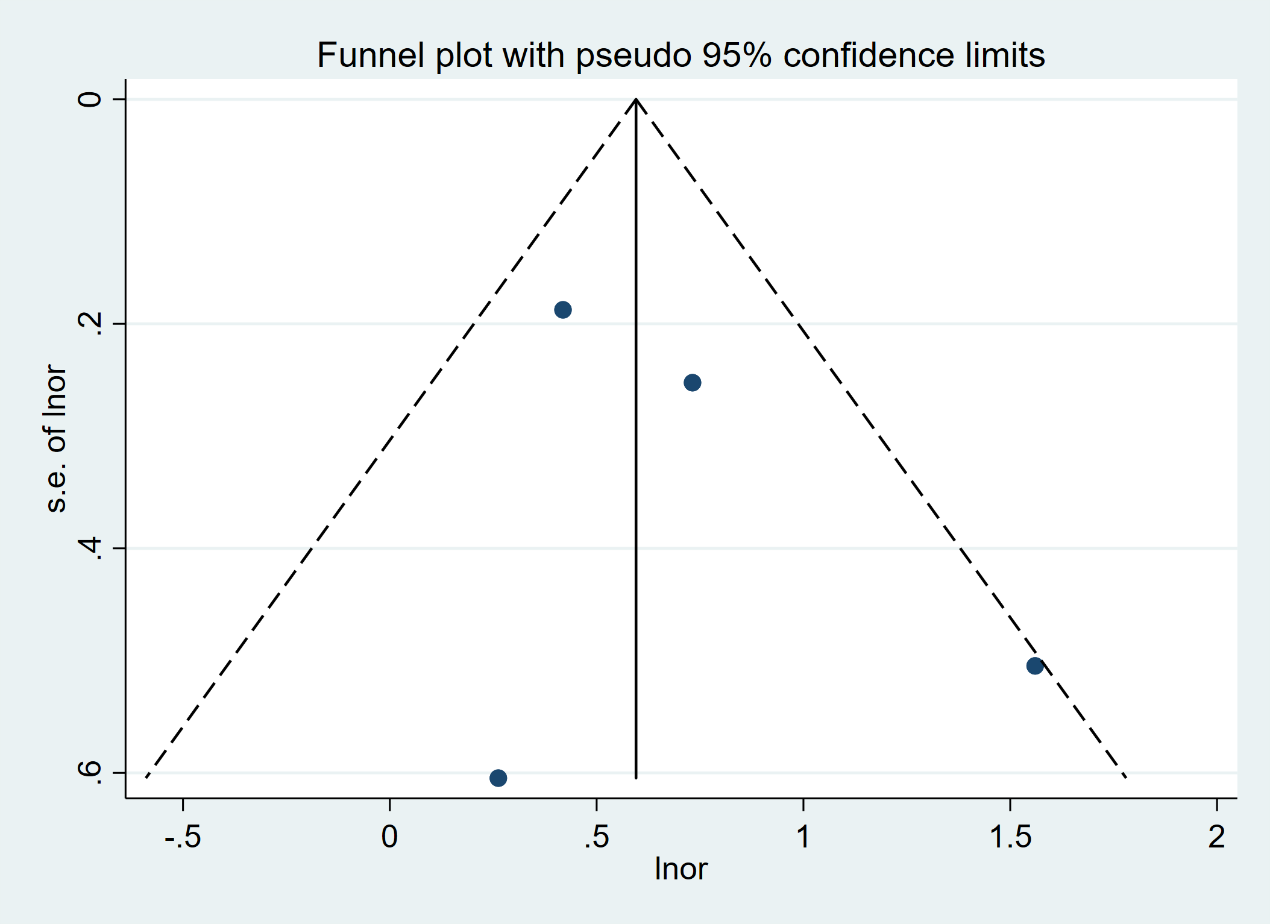
**

**S18Fig. The result of the prevalence of Egger's test**

**
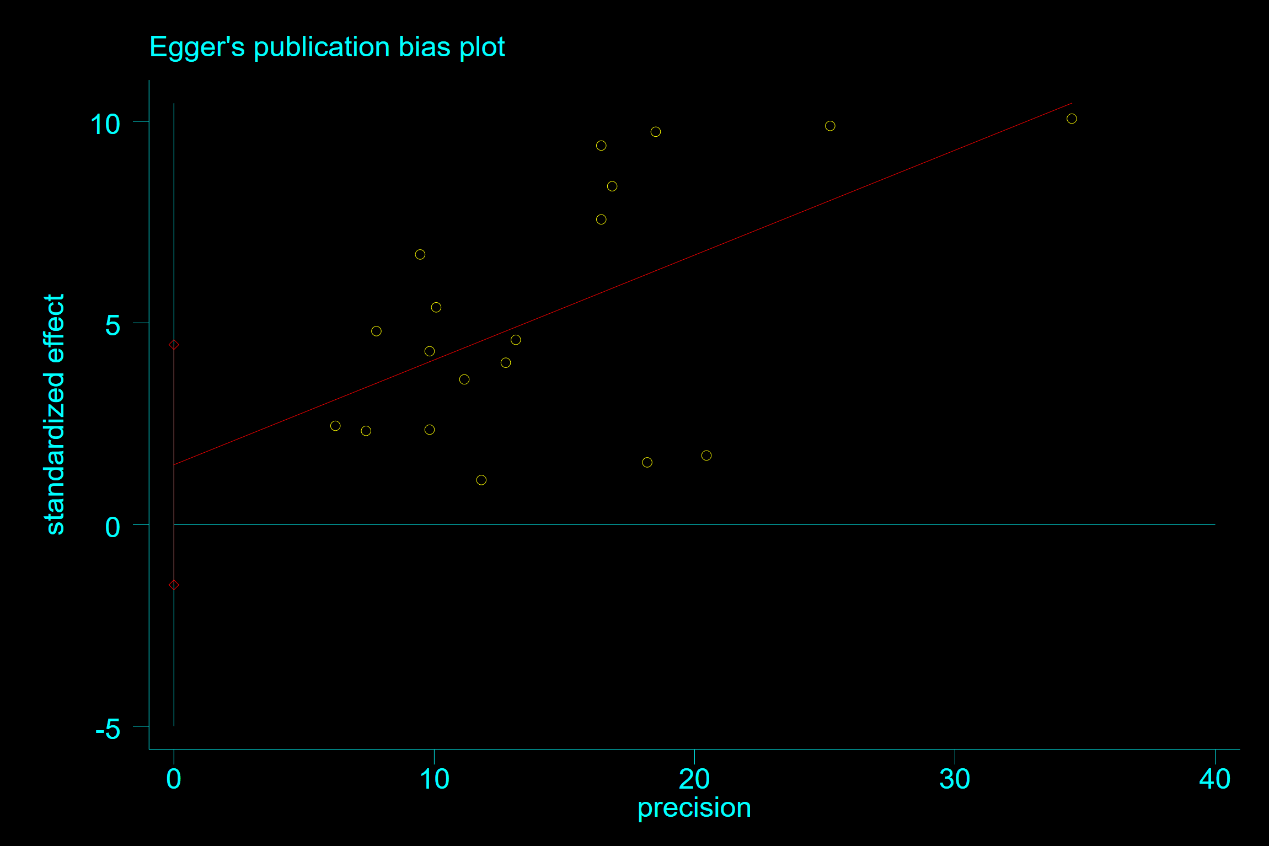
**

**S19Fig. The results of the leave-one-out method of recipient age**

**
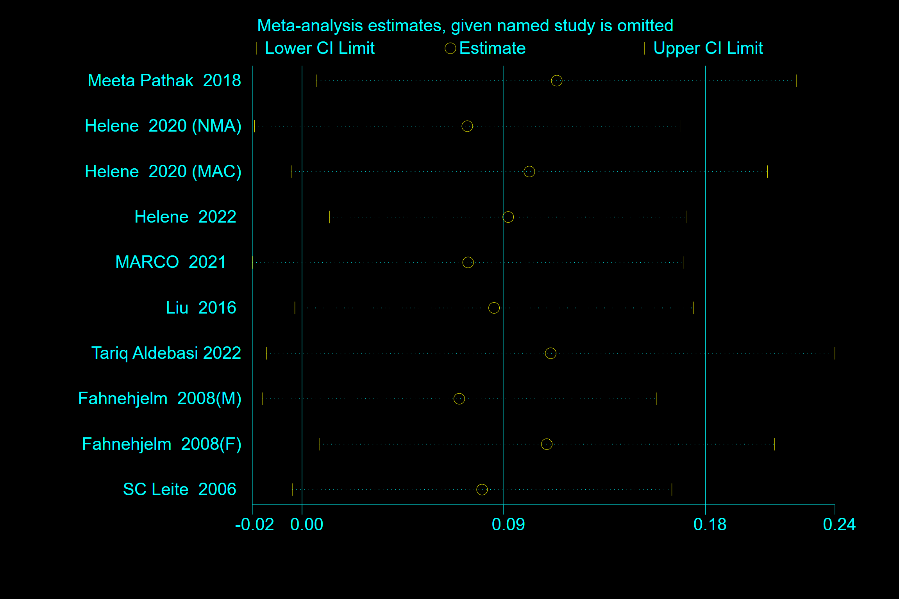
**

**S20Fig. The results of the leave-one-out method of Donor gender**

**
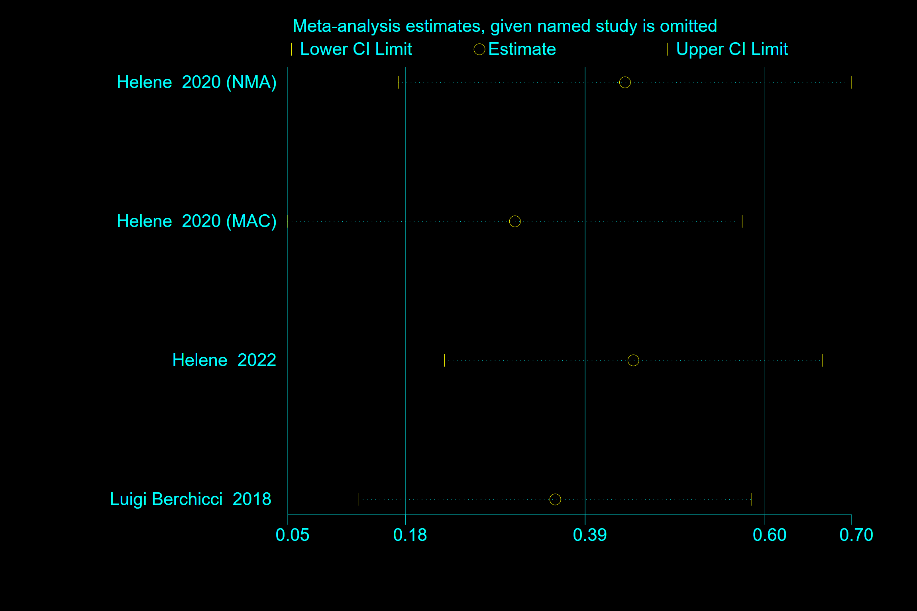
**

**S21Fig. The results of the leave-one-out method of recipient gender**

**
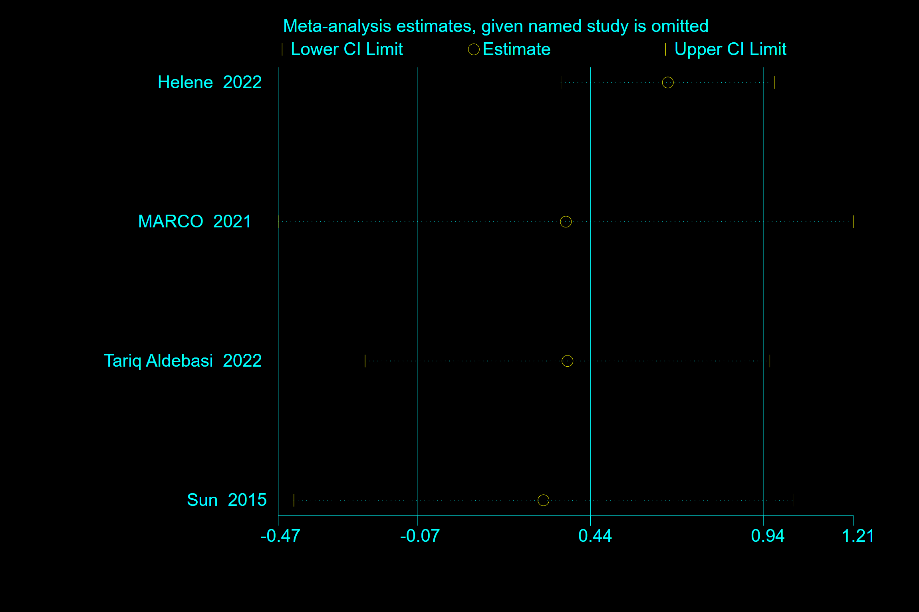
**

**S22Fig. The results of the leave-one-out method of sex matched**

**
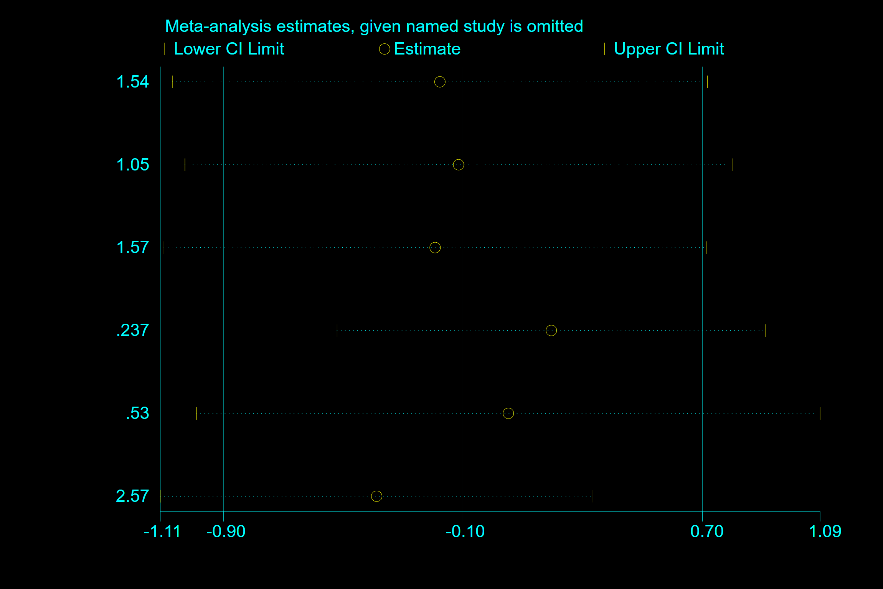
**

**S23Fig. The results of the leave-one-out method of donor type**

**
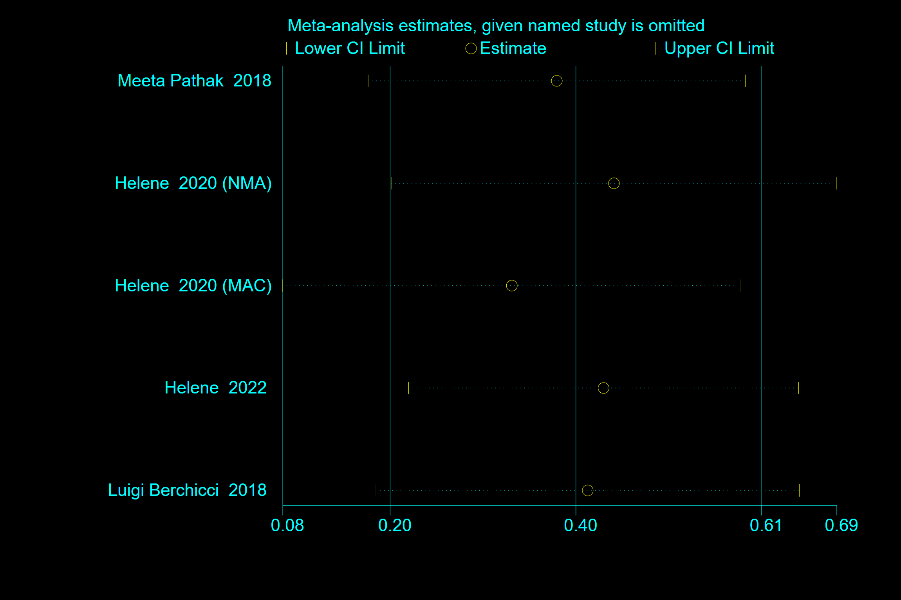
**

**S24Fig. The results of the leave-one-out method of aGVHD**

**
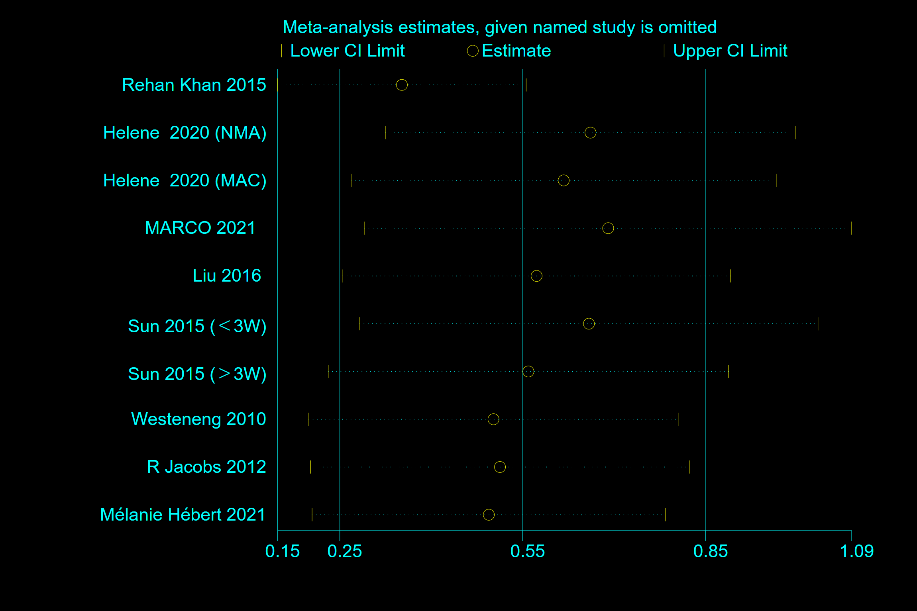
**

**S25Fig. The results of the leave-one-out method of skin GVHD**

**
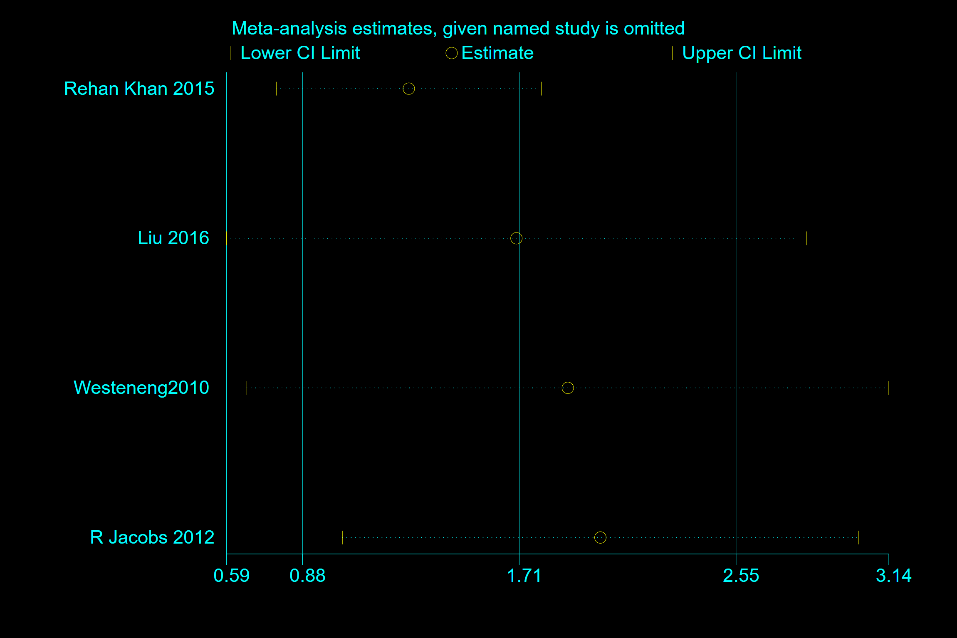
**

**S26Fig. The results of the leave-one-out method of source of stem cells**

**
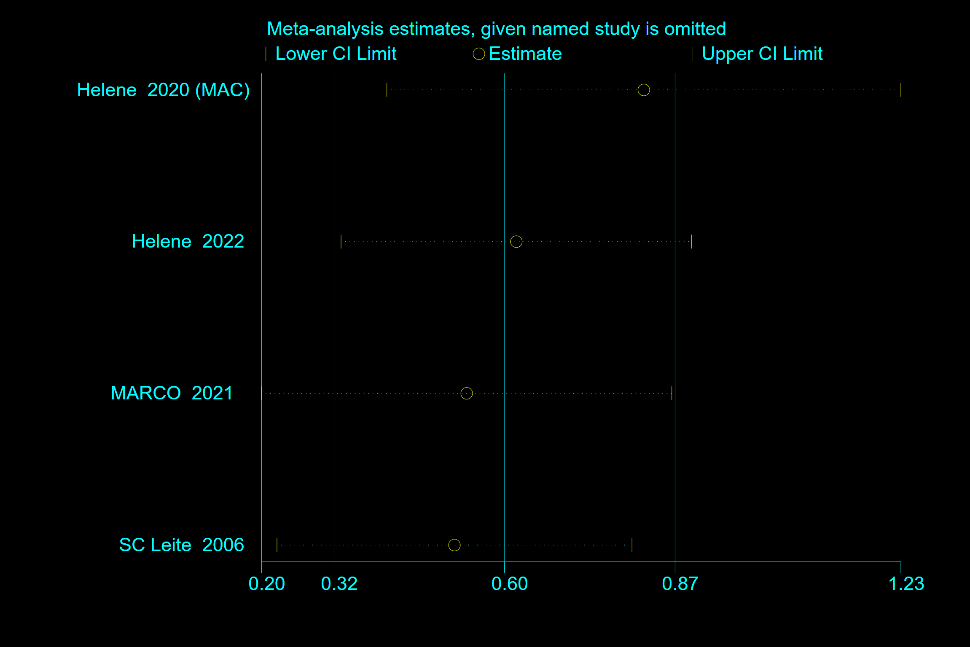
**

**S27Fig. The results of the leave-one-out method of malignant disease**

**
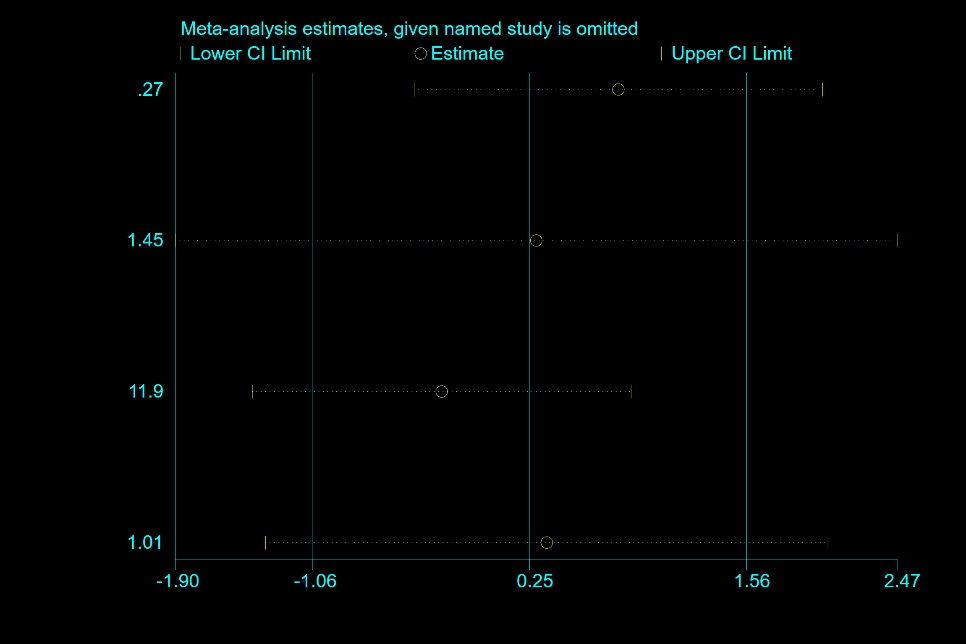
**

**S28Fig. The results of the leave-one-out method of TBI**

**
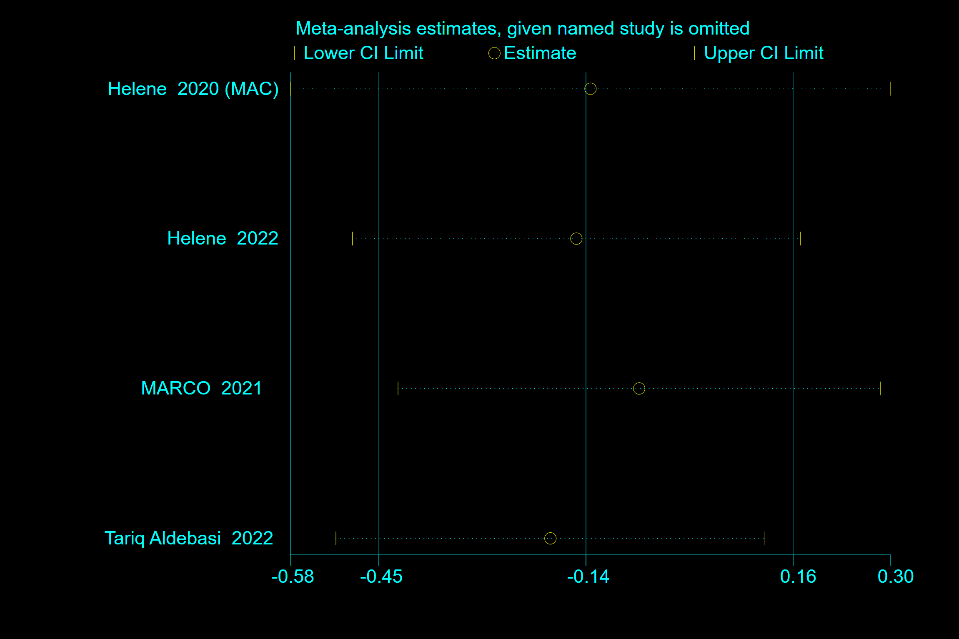
**

**S29Fig. The results of the leave-one-out method of SIT＜10 mm/5 min**

**
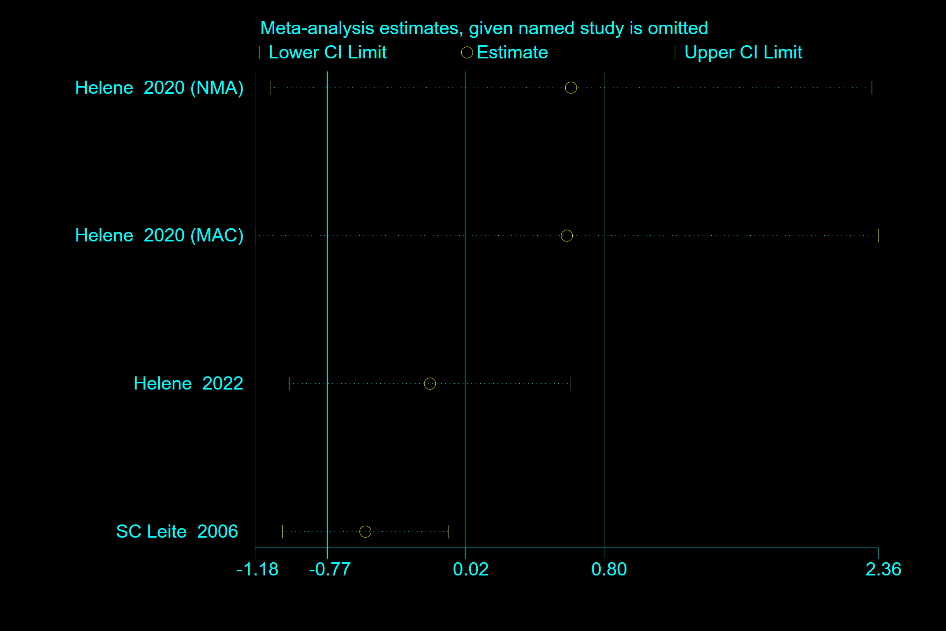
**

**S30Fig. The results of the leave-one-out method of prevalence**

**
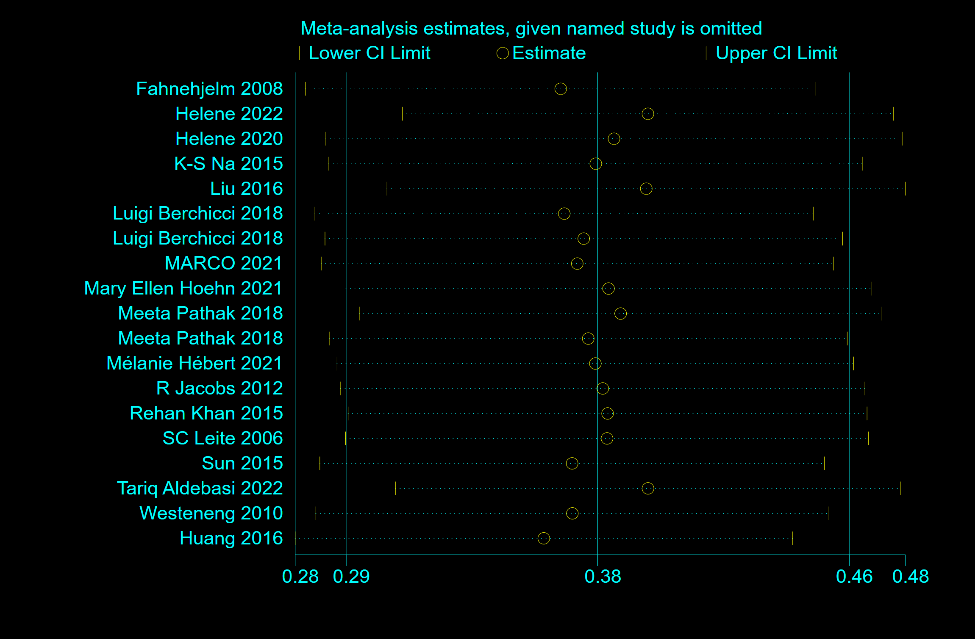
**
